# Macrocephaly and Finger Changes: A Narrative Review

**DOI:** 10.3390/ijms25105567

**Published:** 2024-05-20

**Authors:** Cecilia Lazea, Romana Vulturar, Adina Chiș, Svetlana Encica, Melinda Horvat, Cristina Belizna, Laura-Otilia Damian

**Affiliations:** 1 1st Department of Pediatrics, “Iuliu Hațieganu” University of Medicine and Pharmacy Cluj-Napoca, 400370 Cluj-Napoca, Romania; cecilialazea@umfcluj.ro; 2 1st Pediatrics Clinic, Emergency Pediatric Clinical Hospital, 400370 Cluj-Napoca, Romania; 3 Department of Molecular Sciences, “Iuliu Hațieganu” University of Medicine and Pharmacy Cluj-Napoca, 400349 Cluj-Napoca, Romania; romanavulturar@gmail.com; 4 Cognitive Neuroscience Laboratory, University Babes-Bolyai, 400015 Cluj-Napoca, Romania; 5 Association for Innovation in Rare Inflammatory, Metabolic, Genetic Diseases INNOROG, 30E, Făgetului St., 400497 Cluj-Napoca, Romania; ldamian.reumatologie@gmail.com; 6 Department of Pathology, “Niculae Stancioiu” Heart Institute Cluj-Napoca, 19-21 Calea Moților St., 400001 Cluj-Napoca, Romania; s_encica@yahoo.com; 7 Department of Infectious Diseases and Epidemiology, The Clinical Hospital of Infectious Diseases, “Iuliu Hatieganu” University of Medicine and Pharmacy Cluj-Napoca, 400348 Cluj-Napoca, Romania; melinda.horvat@umfcluj.ro; 8 UMR CNRS 6015, INSERM U1083, University of Angers, 49100 Angers, France; cristina.belizna@wanadoo.fr; 9 Internal Medicine Department Clinique de l’Anjou, Vascular and Coagulation Department, University Hospital Angers, 49100 Angers, France; 10 Department of Rheumatology, Center for Rare Musculoskeletal Autoimmune and Autoinflammatory Diseases, Emergency Clinical County Hospital Cluj, 400006 Cluj-Napoca, Romania; 11 CMI Reumatologie Dr. Damian, 400002 Cluj-Napoca, Romania

**Keywords:** macrocephaly, high forehead, development, overgrowth, interferonopathy, autoinflammatory, autoimmune, inherited metabolic disorders, ciliopathies, infection

## Abstract

Macrocephaly, characterized by an abnormally large head circumference, often co-occurs with distinctive finger changes, presenting a diagnostic challenge for clinicians. This review aims to provide a current synthetic overview of the main acquired and genetic etiologies associated with macrocephaly and finger changes. The genetic cause encompasses several categories of diseases, including bone marrow expansion disorders, skeletal dysplasias, ciliopathies, inherited metabolic diseases, RASopathies, and overgrowth syndromes. Furthermore, autoimmune and autoinflammatory diseases are also explored for their potential involvement in macrocephaly and finger changes. The intricate genetic mechanisms involved in the formation of cranial bones and extremities are multifaceted. An excess in growth may stem from disruptions in the intricate interplays among the genetic, epigenetic, and hormonal factors that regulate human growth. Understanding the underlying cellular and molecular mechanisms is important for elucidating the developmental pathways and biological processes that contribute to the observed clinical phenotypes. The review provides a practical approach to delineate causes of macrocephaly and finger changes, facilitate differential diagnosis and guide for the appropriate etiological framework. Early recognition contributes to timely intervention and improved outcomes for affected individuals.

## 1. Introduction

Congenital anomalies are common, occurring in at least 2–3% of infants and are major drivers of mortality and morbidity [1]. Understanding the etiology of malformations may aid the search for modifiable causes of abnormalities [1]. Recent progress in molecular sciences has revealed, aside from monogenic disorders, a large array of non-Mendelian genetic contributors to this pathology [1]. However, clinical observation is invaluable in guiding the examinations, and the distance from the bench to the bedside is shortened by the astute clinicians who notice subtle disease features.

We aimed to review what follows the diseases which are associated with macrocephaly and finger changes, in the hope of helping the practitioners facing these anomalies that either manifest in infancy or later during development. The manuscript combines elements of both narrative and synthetic reviews, providing an overview of the subject matter by synthesizing information from various sources. The list of rare disorders characterized by the association of “macrocephaly with finger changes” was generated using FindZebra database and subsequently cross-referenced with data from the OMIM database. Furthermore, a search was conducted using PubMed with the following medical subject headings (MeSH terms): (macrocephaly OR megalencephaly OR hydrocephalus) and (finger OR digits OR hands OR upper extremity OR nails). The English language filter was the single one applied in the search. However, single mentions of a feature of interest that were not included in OMIM were excluded.

Macrocephaly (MC) is caused by an increase in the head size, defined as an increased occipitofrontal circumference of above two standard deviations or greater than the 97th percentile corresponding to the age, sex, and gestational age. 

According to Barbier et al. (2013), the mean normal head circumferences by gestational age from 24 to 40 weeks are as follows (in cm): at 24 weeks, it is 22.7 for boys and 22.1 for girls. At 26 weeks, it increases to 24.6 for boys and 23.8 for girls. By 28 weeks, the mean head circumference reaches 26.3 for boys and 25.7 for girls. Advancing to 30 weeks, it measures 28.3 for boys and 27.7 for girls. At 32 weeks, the average head circumference is 30.1 for boys and 29.6 for girls. Continuing to 34 weeks, it grows to 31.9 for boys and 31.5 for girls. By 36 weeks, it reaches 33.5 for boys and 33.1 for girls. At 38 weeks, it is 34.6 for boys and 34.0 for girls. Finally, at 40 weeks, the mean head circumference is 35.2 for boys and 34.6 for girls [2]. References of normal mean head circumference on gestational age (24–40 weeks) are listed in the Supplementary Materials Table S1. When the occipitofrontal circumference exceeds three standard deviations, neurogenetic disorders are usually associated [3,4,5,6]. Single-gene disorders are responsible for some MC cases, but most MC cases have uncertain etiology [7]. 

Megalencephaly (ME) is defined as the enlargement of the brain parenchyma of more than two standard deviations above the age-related mean. ME is caused by the abnormal size or number of dysfunctional neurons and/or glia. Brain development is controlled by multiple signaling pathways involved in proliferation, migration, and organization of neurons and glia (mTOR, Ras/MAPK, and SHH pathways) [4,8,9]. Copy number variations (CNVs), which are an important source of genetic variability, can also be considered causative factors of ME, together with mosaicism and epigenetic mutations [8,9]. Usually, ME is associated with developmental disabilities and is often more syndromic than MC [10]. Autism is often associated with ME or MC. The defective neuronal migration resulting in abnormal laminar positioning of cortical projection neurons, along with the inappropriate synaptic pruning and arborization, and with the consequently increased dendrite number and size all possibly connect MC with autism [3].

HC is a common condition caused by physical or functional obstruction of the cerebrospinal fluid (CSF) flow, leading to active distention of the ventricular system [11,12,13]. CSF is produced by the choroid plexi, passes through the ventricular system to the subarachnoid space, and it is absorbed into the venous sinuses and undergoes systemic circulation. HC has both genetic and environmental causes and can be congenital or acquired. When acquired, HC is considered a complication of various conditions such as hemorrhage, infection, neoplasia, or medication taken during pregnancy acting upon a structurally normal brain. Patients with HC can present other physical abnormalities or can have predominantly brain anomalies [11]. *L1CAM* mutations are the main genetic causes of isolated HC [3,5,11]. 

MC can be present at birth or can occur later during postnatal growth. MC, affecting up to 5% of children, is often benign familial or due to benign external HC and may be associated with over 200 genetic disorders or other progressive etiologies [3]. 

MC is attributed to the increase in size of any of the cranium components (brain, cerebrospinal fluid, blood, or bone) or to increased intracranial pressure, The hypertrophic or hyperplastic structure involved may give a clue on the underlying pathology. The general causes of MC with examples are listed in Supplementary Materials Table S2. 

The presence of neurocutaneous features may point to neurofibromatosis or Legius syndrome, tuberous sclerosis, cardiofaciocutaneous syndrome, Costello syndrome, LEOPARD, Gorlin (nevoid basal carcinoma syndrome), Noonan syndrome etc. The MC and other overgrowth features, in the presence of concomitant vascular changes, may suggest the PI3KCA-Related Overgrowth Syndrome (PROS) or other diseases associated with activation of the PI3K/AKT/mTOR intracellular pathways, such as the CLOVES syndrome (Congenital Lipomatous Overgrowth, Vascular Malformations, Epidermal Nevis, Spinal/Skeletal Anomalies/Scoliosis), Klippel–Trenaunay syndrome, Proteus syndrome, Megalencephaly–Polymicrogyria–Pigmentary Mosaicism syndrome and others. Other examples of overgrowth syndromes evolving MC are the syndromes Sotos and Weaver, Beckwith–Wiedemann, Simpson–Golabi–Behmel, or Cowden’s, and macrocephaly, dysmorphic facies, and psychomotor retardation (MDFPMR) syndrome [3,5,11,14,15,16]. 

The metabolic causes of MC include organic acid disorders (Glutaric acidemia type I, type II D2-hydroxyglutaric aciduria), lysosomal storage diseases (mucopolysaccharidosis, gangliosidosis, Krabbe disease), peroxisome biogenesis disorders (Zellweger/cerebrohepatorenal syndrome), and leukoencephalopathies (Alexander disease, Canavan disease, megalencephalic leukoencephalopathy with subcortical cysts). 

Excessive CSF may increase the size of the cephalic extremity, causing HC, either obstructive (such as in brain tumors, Chiari malformation, Dandy–Walker syndrome malformation, aqueductal stenosis) or communicative, due to deficient CSF resorption or altered blood circulation within the brain. The benign enlargement of the subarachnoid space or cerebral hemorrhages may produce similar effects. Increased intracranial pressure (in infections, metabolic disorders, pseudotumor cerebri, intoxications etc.) may result in MC [3,5,11,14,15,16].

Bone disorders involving the bone marrow (as in thalassemia) or the bone structure (in rickets), skeletal dysplasia (achondroplasia, cleidocranial dysostosis, osteogenesis imperfecta, hyperphosphatasia, osteopetrosis, osteopathia striata with cranial sclerosis and others) may be associated with MC. 

The genetic mechanisms underlying the development of head bones and of extremities are complex. Excessive growth may be caused by alteration of the complex interaction between the genetic, epigenetic, and hormonal factors orchestrating human growth [17].

The etiopathogenesis of many overgrowth syndromes has been recently clarified. The overgrowth may be generalized or segmental, interesting one or a few regions of the body. The latter is often due to overactivating mutations of PI3K/AKT/mTOR. Sometimes the mutations appear in a mosaic manner, and the genetic diagnosis is not straightforward. A head circumference of ≥98%, even in the absence of other findings, may be associated with autism or intellectual disability [17]. The PI3K/AKT/mTOR pathway is closely related to the RAS/MAPK pathway [18]. In some overgrowth syndromes, variants in epigenetic regulators are associated with disease occurrence, such as variants of histone methyltransferases NSD1 in Sotos syndrome and EZH2 in Weaver syndrome [19,20]. Other extracellular modulators may result in certain skeletal dysplasias with skull and extremities involvement [21].

Some of the MC cases are related to the premature fusion of one or more cranial sutures of the skull called craniosynostosis [22]. Cranial sutures, fibrocellular structures separating the skull bones plates, enable the skull growth in coordination with the developing brain [22]. The premature fusion of a suture may allow the compensatory growth of other parts to accommodate the enlarging brain, resulting in specific deformities [22]. Not all craniosynostoses evolve with MC, some of them do not change in the head size or shape, while the others might result in brachycephaly, or other vault morphology defects [23]. In the genetics of craniosynostoses, the fibroblast growth factor receptors (FGFR) play an important role [24]. Genes associated with skeletal diseases and ME, such as FGFR-associated craniosynostosis syndromes, RAS pathway-associated syndromes and PI3K-AKT pathway associated ME syndromes have also been involved in HC pathogenesis. Growth factors (like epidermal, vascular, insulin-like EGF, VEGF, IGF3, etc.) influence the pathways in some overgrowth syndromes as well [19].

During development, the skull bones grow in a spatially and temporally coordinated fashion [25]. Premature fusion of one or multiple cranial sutures may lead to craniosynostosis, leading to head size or morphology alterations [23]. About 15% of the craniosynostosis cases are syndromic [23]. Calvaria bones have a dual embryonic origin, namely from the cranial neural crest cells and from paraxial mesoderm and ossified directly through intramembranous ossification [25]. The frontal and parietal calvaria bone origin from the supraorbital arch mesenchyme, which acts as an “organizer” for the upper skull bones [25]. Angiogenesis is necessary for intramembranous ossification and for enchondral bone formation [23].

The signaling pathways for the calvarial bones include the Wnt signaling pathway and its effectors such as TWIST1, and transcription factors such as the forkhead-domain-containing Fox family and Twist1 (basic-helix loop-helix transcription factor), along with transcription factors such as Msh Homeobox1 (Msx1), and 2 (Msx2), Runt Related Transcription factor 2 (Runx2), and Osterix (Osx/Sp7). Other factors involved in the frontal and parietal bone development are the Transforming Growth Factor Receptor beta II (TGF beta RIi) or Sp8 (Specificity Protein 8, the FGF8 regulator) [25].

For the sutures, major cellular signaling pathways (WNTs, BMPs, FGFs and others) produce a complex set of instructions for the undifferentiated mesenchymal cells to become osteoblasts lineage cells and then to progress to osteocytes [23]. 

Premature suture closure may result from disruption in the multistep and finely tuned process involving the development, influenced by genetic, environmental, and other intervening factors [23]. The core set of transcription factors and signaling pathways involved in the skull bone development may also be disrupted into the acral appendicular skeleton development. 

In the extremities, proliferating chondrocytes in the epiphyseal plate of long bones underlie the skeletal growth [17]. Chondrocyte proliferation in the growth plate is increased by the Indian Hedgehog (IHH), which stimulates PTH-related protein (the IHH-PTHrP pathway) and bone morphogenetic protein (BMP) and is decreased by the FGF–FGF3 pathway [17]. Chondrocyte hypertrophy is inhibited by the IHH-PTHrP pathway and stimulated by thyroid hormones through the Wnt4 (Wingless-int4)-beta-catenin pathway [17]. Both chondrocyte proliferation and hypertrophy are stimulated by the growth hormone (GH) insulin-like growth factor 1 (IGf1) pathway [17].

## 2. Causes of Macrocephaly and Finger Changes

In the presence of MC, any other morphological changes, even subtle and discreet, may provide clinical clues for the clinician to identify a certain pathology. The differential diagnosis of finger changes in the context of MC is vast.

The many acral changes possibly involving the hands can constitute valuable hints for the examiner. Polydactyly may be found along with enlarged head in several syndromes (Table 1). Camptodactyly refers to flexural deviation in the proximal interphalangeal joint, while clinodactyly to the deviation in the radioulnar plan distal to the metacarpophalangeal joint [26]. Finger tapering is defined as the gradual reduction in girth of the digit from proximal to distal [27]. Many of the conditions evolving with the cranial and acral changes are complex syndromes with pluriorganic involvement, including cerebral, with developmental, neurologic, and psychiatric features (detailed elsewhere). 

### 2.1. Skeletal Dysplasias

Skeletal dysplasias are important causes of familial MC [8]. Achondroplasia evolves with ME, and it gives rise to specific facial features including frontal bossing and midface hypoplasia, thoracic kyphosis, lumbar lordosis, short stature, brachydactyly, and “trident hand deformity” [98]. In the nail-patella syndrome, a high forehead with receding hairline, a lean trunk, and hypoplastic or absent nails are also noted [98]. Pycnodysostosis, from the osteopetrosis disease spectrum, probably the disease of Toulouse–Lautrec, may evolve with MC and manifest in frontal bossing, short stature, and the acro-osteolysis of the terminal phalanges [69].

In lysosomal storage disorders, such as mucopolysaccharidoses, skeletal abnormalities known as dysostosis multiplex often accompany macrocephaly. These abnormalities may include dolichocephaly, facial deformities, thoracic abnormalities, proximally pointed metacarpals, and broad, bullet-shaped phalanges [8,83,84,98,116]. Beta-thalassemia evolves with macrocephaly, alongside “tower skull” due to ectopic hematopoiesis, lateral malar prominence, kyphosis, and decreased spinal height, sausage-like fingers and sometimes hypercoagulability, leg ulcers, and vascular changes [67,117,118,119]. Similar changes have been rarely described in sickle cell diseases and hereditary spherocytosis, and in uncorrected cyanotic heart disease, due to reactive bone marrow expansion [113].

### 2.2. Inherited Metabolic Disorders 

Inherited metabolic disorders comprise various conditions, each with unique clinical presentations. Several of these conditions, including Mucopolysaccharidoses, Gangliosidosis, Alpha-mannosidosis type I, and Peroxisome Biogenesis Disorders, are characterized by MC and finger changes [16]. Mucopolysaccharidoses (MPS) represent a group of lysosomal storage disorders (LSDs), characterized by the accumulation of glycosaminoglycans (GAGs) due to deficiencies in specific enzymes involved in their degradation. Alongside skeletal abnormalities and developmental delay, individuals with MPS may present MC, attributed to the accumulation of GAGs in the brain and subsequent HC. Gangliosidosis type I, a subtype of GM1 gangliosidosis, is caused by a deficiency in the enzyme named β-galactosidase, leading to the accumulation of GM1 ganglioside primarily in the central nervous system. MC is a common finding in affected individuals, often accompanied by typical facial features and skeletal abnormalities. Finger changes, such as claw hand deformities, can also occur due to progressive skeletal dysplasia. Alpha-mannosidosis type I is a rare autosomal recessive disorder resulting from a deficiency in the enzyme, alpha-mannosidase. MC may manifest in affected individuals, likely due to cerebral edema and HC. Additionally, skeletal abnormalities such as dysostosis multiplex can lead to distinctive changes in the fingers, including the shortening and thickening of the digits.

Peroxisome Biogenesis Disorders (PBD) are a group of autosomal recessive diseases characterized by impaired peroxisome assembly and function. Some forms of PBD may present with MC, likely due to associated brain anomalies. Finger changes may also occur, with variability depending on the specific subtype of PBD and its clinical features [16,84].

### 2.3. Overgrowth Syndromes

**Figure 1:**
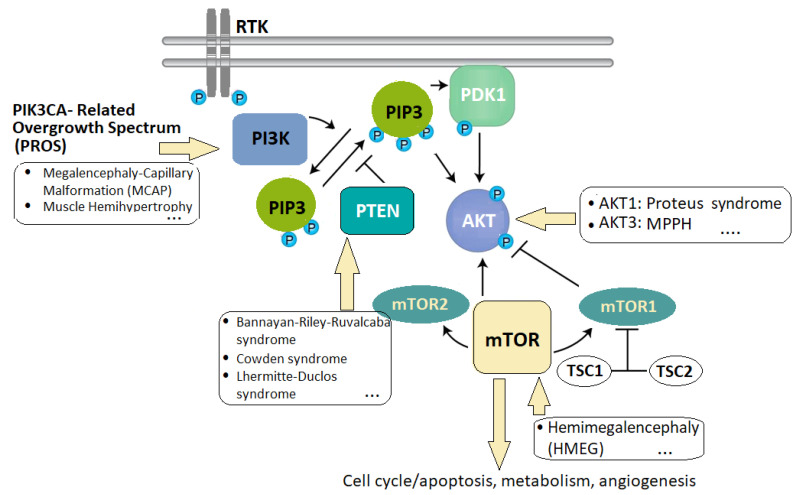
The PI3K–AKT pathway, a critical signaling cascade within cells that regulates various cellular processes, including cell cycle, metabolism, proliferation, and survival (image based on [123,124]). Legend: The mTOR (mammalian target of rapamycin) pathway, intersecting with PI3K–AKT, is linked to overgrowth disorders like Proteus syndrome and MCAP (macrocephaly–capillary malformation) syndrome. Dysregulated PI3K–AKT signaling, involving PDK1 (phosphoinositide-dependent kinase, RTKs (receptor tyrosine kinases), and PI3K activation, leads to abnormal cell growth and proliferation. Dysregulation manifests in various disorders such as PROS (PIK3CA-related overgrowth spectrum), HMEG (hemimegaloencephaly), MCAP, and MPPH (megalencephaly–polymicrogyria–polydactyly–hydrocephalus) syndrome. Mutations in *TSC1* and *TSC2* (tuberous sclerosis complex 1 and 2) further contribute to overgrowth disorders, highlighting genetic complexity.

The overgrowth syndromes are a heterogenous group of rare disorders characterized by excessive growth, either generalized or segmental, associated with MC and often other additional features [120]. The general term of PROS (PIK3CA-Related Overgrowth Spectrum) was agreed upon to cover all known and emerging clinical diseases associated with somatic mutations in *PIK3CA*. For example, within this spectrum, various entities have been described with different degrees of overgrowth associated with vascular anomalies (see Figure 1). Overlap syndromes are best understood as the clinical picture of a spectrum of diseases rather than the result of an enumeration of clinical criteria. Despite being caused by the same mutations in *PIK3CA*, the clinical course and outcomes of neonates with CLAPO syndrome (OMIM 613089—associating capillary malformation of the lower lip, lymphatic malformation of the face and neck, asymmetry, and partial/generalized overgrowth) and MCAP (megalencephaly–capillary malformation) syndrome are different [121]. In CLAPO syndrome, overgrowth is not always obvious, MC is absent, and involvement tends to be segmental rather than generalized. Facial asymmetry often stems from vascular factors. It is crucial to differentiate apparent asymmetry from genuine overgrowth due to hyperplasia or hypertrophy, as seen in CLAPO and PROS syndromes [122]. 

HC may belong to various dysmorphic syndromes including RASopathies, disorders caused by germline mutations of the RAS/MAPK signaling pathway, or its regulators [3,105,107,125]. Glomus tumors of the fingers (benign neoplasms that arise from the glomus body, a specialized thermoregulatory shunt which is concentrated in the fingers and toes) are associated with neurofibromatosis and their appearance is determined by the loss of neurofibromin function [126,127]. In neurofibromatosis type 1 (NF1), a common AD syndrome with variable expressivity, MC may be due to ME and is frequently associated to a short stature [9]. Fingers in NF1 may be affected by neurofibromas, glomus tumors, bone enlargement, pseudarthrosis of the forearm or hand bones, etc. [128]. Legius syndrome has similar cutaneous changes as NF1 and may have MC, but other non-pigmentary disease features of NF1 are lacking [129]. 

Cardiofaciocutaneous syndrome (CFC) is characterized by a range of features including cutaneous abnormalities, craniofacial dysmorphism, gastrointestinal dysmotility, and cognitive impairment [130]. Individuals with CFC often present with MC, accompanied by bitemporal narrowing, a small chin, palpebral ptosis, downslanting eyes, epicanthic folds, sparse or absent eyebrows, and rare hair [130]. Additionally, palmo-plantar hyperkeratosis, scaly skin, hemangioma, and multiple nevi may be observed [130]. In Costello syndrome, MC is accompanied by coarse facial features, downslanting palpebral fissures, bulbous nose, full cheeks, large mouth, and nasal papillomas, cardiovascular abnormalities, and increased cancer risk. Acral changes include ulnar hand deviation, nail dystrophy, cutis laxa, and diffuse skin hyperpigmentation [130]. 

Nevoid Basal Cell Carcinoma Syndrome (Gorlin–Golitz syndrome or Gorlin syndrome) is marked by various signs of abnormal development, including macrocephaly, mild hydrocephalus, intracranial calcification, and EEG abnormalities. Anomalies in the ribs and vertebrae, brachydactyly, short fourth metacarpal, short thumb terminal phalanx, cleft lip or palate, along with multiple basal cell carcinomas and skin epidermal cysts, calcified dural folds, keratocysts in the jaws, ovarian fibromas, medulloblastomas, lymphomesenteric cysts, fetal rhabdomyomas were also described [31,131].

Sturge–Weber syndrome manifests with an abnormality in the brain’s blood vessels (leptomeningeal angiomatosis), predominantly affecting the posterior parietal and occipital lobes. Common features include MC, facial and choroidal hemangiomata, seizures, and glaucoma [132].

The PTEN Hamartoma tumor syndrome (PHTS), due to mutations in the PTEN (phosphatase and tensin homologue deleted on chromosome Ten) gene, evolves with macrocephaly, vascular malformations, and hamartomas [133]. Lhermitte–Duclos syndrome or dysplastic cerebellar gangliocytoma is part of the PHTS spectrum and may be associated with MC or ME, syringomyelia, polydactyly, and malignancies, and sometimes within Cowden’s syndrome [134]. Pretzel syndrome, or the PMSE (polyhydramnios, ME, and symptomatic epilepsy) results from mutations in the STRAD-alpha gene and generally evolves with important joint hypermobility, allowing the development of abnormal joint postures (hence the name “pretzel”) [102]. Other syndromes with macrocephaly and skin changes (most often due to vascular abnormalities) are Klippel–Trenaunay, MCAP, megalencephaly–polymicrogyria–polydactyly–hydrocephalus (MPPH) or CLOVES (Congenital Lipomatous Overgrowth, Vascular Malformations, Epidermal Nevis, and Spinal/Skeletal Anomalies/Scoliosis) [78,135,136]. 

The Sotos and Weaver syndromes, overgrowth syndromes produced by germline mutations in the *NSD1* and *EZH2* genes, respectively, encode histone methyltransferases and have considerable clinical overlap with MC, giving rise to a high, broad forehead, and prominent chin. Moreover, mostly in Weaver’s syndrome, camptodactyly and deep-set nails evolving into a boutonniere deformity in adulthood are reported [19]. Malan’s syndrome has a similar appearance with MC, a long and narrow triangular face, prognathism, long hands, advanced bone age and scoliosis, and sometimes, aortopathy [20].

Robinow’s syndrome may associate MC with frontal bosses, midface hypoplasia and brachydactyly [65,66]. Mutations of *MED12* on the chromosome X at q13 causes X-linked intellectual disability, with four different phenotypes [137]. Of these, in the Lujan–Fryns syndrome, also called X-linked intellectual disability with marfanoid habitus syndrome, MC may be present along with distinct facial dysmorphism, nasal voice, long slender fingers, arachnodactyly, sandal gap, and behavior problems [138]. In the Opitz–Kaveggia syndrome, another MED12-associated allelic disease, the clinical features are similar, but the fingers and toes are broad [137]. 

Some ciliopathies such as the short-rib thoracic dysplasia 8 with or without polydactyly (SRTD8), due to *WDR60* mutations, evolve with MC, and give rise to skin changes, along with renal and neurological involvement [30]. The rare Adams-Oliver syndrome, a multisystemic disease, may present with or without cutis marmorata telangiectatica congenita and evolves with the scalp and sometimes, skull bone abnormalities and terminal limb defects, including abnormally short fingers and toes with small or absent nails [82]. The Cole-Carpenter syndrome is considered a severe form of osteogenesis imperfecta [96].

### 2.4. Congenital Infections

Congenital cytomegalovirus (CMV) infection may affect the CNS and result in HC, temporal cysts, delayed myelination, microcephaly (MiC) or sometimes, MC, with a whole plethora of cerebral or sensorineural abnormalities [63]. Associated finger changes may consist of brachydactyly with rudimentary fingernails, finger agenesis, and syndactyly [64,139]. 

Parvovirus or rubella may result in HC and cerebral vasculitis/vasculopathy [63].

### 2.5. Autoimmune and Autoinflammatory Diseases

HC is rarely described in autoimmune diseases, including systemic lupus erythematosus, juvenile idiopathic arthritis, or systemic sclerosis [140]. MC, mainly frontal bossing, and HC may belong to the clinical spectrum of autoinflammatory diseases, mostly in criopyrinopathies such as CINCA/NOMID or Muckle–Wells syndrome [103]. Moreover, prominent frontal bossing, triangular face, and hypertelorism are encountered in mevalonate kinase deficiency [104]. Urticarial-like and other types of rashes may occur in these diseases. 

Fingertip skin lesions including chilblain-like erythema, vasculitis, Raynaud’s phenomenon, ulceration, or necrosis may occur in systemic lupus erythematosus or in vasculitis (including in the adenosine deaminase-2 deficiency, DADA2, in which skull involvement is not commonly described) [141]. Also, all the above skin lesions, as well as the red scaly lesions suggesting psoriasis, or cold-induced severe ulcerative lesions of fingers, toes, or ears in a child with systemic inflammation may be clinical “red flags” suggesting an interferonopathy [100,142]. Many diseases in this group have overlapping clinical features, including the CNS involvement [100]. 

Interferons (IFNs) are molecules involved in the first defense against pathogens [100]. Viral and bacterial pathogens are sensed by pattern recognition receptors, stimulating intracellular pathways with IFN secretion [143]. The interferonopathies result either from excessive stimulation, or to defective regulation of the type I IFN pathways [143].

The constitutive hyper-activation of type I IFN responses may present as early-onset, severe, and atypical rheumatic diseases [100]. The conditions include Aicardi–Goutières syndrome (AGS), familial chilblain lupus, monogenic forms of lupus, spondyloenchondrodysplasia with immune features (SPENCD), the proteasome-associated autoinflammatory syndromes (PRAAS), the IFN-stimulated gene-15 deficiency, Singleton–Merten syndrome (SMS) and its atypical presentation, the stimulator of IFN genes (STING)-associated vasculopathy with onset in infancy (SAVI), and the group is rapidly expanding [100,109,142]. Along with systemic inflammation, some of these diseases may present with skeletal involvement and dysmorphic features, and the clinical phenotypes may overlap. Most interferonopathies evolve with MiC, after an early-onset cerebral inflammation resulting in calcification. As for MC, a broad forehead or high hairline were nevertheless described in a few such conditions [70,71,73,74,75,76,77,78,79,80]. The interferonopathies evolving with MC, such as SMS, atypical SMS, or Tenorio syndrome, are transmitted as autosomal dominant (AD) with variable expressivity and incomplete penetrance [70,92,93,95,97,100,109,110,142,144].

Gain-of-function mutations of *IFIH1*, encoding the cytosolic double-strand RNA receptor MDA5, results into a heterogeneous spectrum of phenotypes, and chilblain-like lesions, SMS, AGS and SMS/AGS syndromes overlap, while neurologic features and clinical non-penetrance have been reported within the same family [95,97,109]. SMS is an extremely rare sporadic or inherited multisystem disorder with highly variable expression [95,97,109]. Typical facial features include high anterior hairline, broad forehead, thin upper vermillion, or smooth philtrum [95]. The hands may show acro-osteolysis and/or red, scaly, psoriasis-like rash mainly involving the distal fingers [95]. SMS is characterized by dental dysplasia, progressive calcification of the thoracic aorta and main arteries with stenosis, osteoporosis with fractures of the skull, long bones of arms and legs, and expansion of the marrow cavities in the hand and feet bones. Other patients may have delayed growth, abnormal joints ligaments, hips, and feet malformations, generalized muscle weakness or glaucoma [100]. The atypical SMS (due to *DDX48* mutations) has similar characteristics, but without the dental features [100]. AGS, the typical interferonopathy, evolves with an early-onset infectious encephalitis-like syndrome with fever and neuroinflammation, with secondary cerebral atrophy, calcifications, and MiC, but MC and short trunk have also been described [110,143].

The ubiquitin-specific peptidase 18 (USP18) deficiency due to *USP18* mutations, transmitted AD or AR, may evolve with HC, brain malformation, and systemic inflammation [100,142,144]. In spondyloenchondrodysplasia (SPENCD), transmitted AR, due to mutations of *ACP5* (encoding the tartrate-resistant acid phosphatase 5), skeletal deformity with short stature, platyspondyly, and enchondromas, including cranio-facial and hand deformities, are found along with immune dysregulation, including Sjogren syndrome, SLE and vasculitis features [92,93,94].

MC was also described in 8% of the neonatal lupus erythematosus cases associated with anti-Ro and anti-Ro52 antibodies [114]. Tenorio syndrome, a rare overgrowth syndrome evolving with MC and/or large forehead, neurodevelopmental disease, and systemic inflammation, including Sjogren’s syndrome features, is due to *RNF125* mutations encoding an E3 ubiquitin resulting in dysregulation of several cellular pathways, including that of PI3K–AKT and IFN, respectively [70]. MC was also reported with PTEN mutations in lupus, cutaneous vasculitis, and Cowden’s syndrome with autoimmune features [115,145,146]. Of interest, some RASopathies may also have common pathways with interferonopathies, such as the overgrowth–macrocephaly–facial dysmorphism syndrome, associated with *RNF135* mutations encoding Riplet, a co-receptor of the pattern recognition receptor RIG-I [147,148]. 

A synopsis of the complex differential diagnoses of finger changes in the context of MC is presented in Table 2.

## 3. Clinical Approach and Therapies

**Figure 2:**
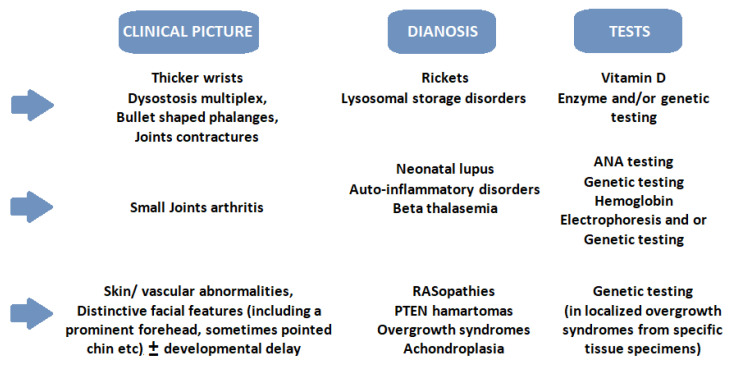
A simplified practical approach to the most frequent causes of macrocephaly and finger changes. Legend: ANA—antinuclear antibodies; PTEN—phosphatase and tensin homologue deleted on chromosome 10.

In the presence of MC and the suspicion of an overgrowth syndrome (see Figure 2), apart from a detailed history, physical examination and imaging studies, the approach should include blood analyses, such as IGF-1, thyroid assessment including free T4, TSH, along with assessment of bone age [17]. 

Nevertheless, genetic testing plays a pivotal role, with next-generation sequencing (NGS) emerging as a transformative tool. NGS enables the screening of a vast array of genetic variations associated with these syndromes, facilitating precise diagnoses. Therapeutically, management strategies are tailored to individual needs, focusing on addressing specific symptoms and complications. This may encompass a multidisciplinary approach involving neurologists, geneticists, and other specialists to provide comprehensive care. Aside from chromosomal microarray analysis, with the advent of NGS, the ability to identify underlying genetic causes has significantly improved, paving the way for more targeted and effective therapeutic interventions for these complex syndromes.

Genetic testing is paramount in identifying underlying syndromes, keeping in mind that negative testing does not rule out a certain syndrome, mainly in, but not limited to, diseases evolving with mosaicism or with somatic mutations. For instance, in some overgrowth syndromes such as PIK3CA-related overgrowth spectrum (PROs), even advanced genetic testing such as next-generation sequencing may be negative when performed from blood samples, and not from the affected tissue [18].

The existence of reciprocal syndromes where deletions may result in macrocephaly while duplications of the same genomic region may lead to microcephaly is a fascinating aspect of genetic variation. These syndromes underscore the delicate balance of gene dosage and expression in neurodevelopment. Malan syndrome, proximal 19p13.3 syndrome, 1q21.1 region anomalies, and NSD1 region aberrations exemplify this phenomenon. Understanding the molecular mechanisms underlying these reciprocal effects can provide important insights into the intricate regulation of brain growth and development. Such knowledge is invaluable for both clinical diagnosis and therapeutic interventions aimed to mitigate the neurological impacts of these genetic alterations. Recognizing a certain disease allows for identification and proactive treatment wherever possible for complications such as an increased cancer susceptibility or others (for instance, Beckwith–Wiedeman syndrome may be associated with hypoglycemia, Malan syndrome with aortopathies, etc.) [17]. 

The potential therapy largely depends on the identification of the underlying cause. Mechanistically, HC may be treated with shunts. Specific surgical procedures (debulking, ray resection, epiphysiodesis, reconstruction or other surgical procedures have been employed for the correction of overgrowth tissue in the extremities, involving the bone, nerve or fibroadipose tissue [168].

Rehabilitation therapy, with functional improvement or at least preservation, is advisable in all cases. Lhermitte–Duclos syndrome and other diseases evolving with partial ME are treated surgically in selected cases. 

In LSDs, where non-degraded substances build up in lysosomes due to mutations in genes encoding lysosomal proteins, the treatment options include the following: enzyme replacement therapy (ERT), substrate reduction therapy (SRT), pharmacological chaperones (PCs), hematopoietic stem cell transplantation (HSCT), and gene therapy (GT). ERT involves administering deficient lysosomal enzymes and has been approved for Gaucher, Fabry, and Pompe diseases, late infantile neuronal ceroid lipofuscinosis type II, acid lipase deficiency, alpha-mannosidosis, and mucopolysaccharidoses (MPS) type I, II, IVA, VI, and VII. SRT inhibits substrate synthesis enzymes. Furthermore, it shows great potential for treating LSDs involving neurological issues, but substantial advancements depend on the ability of new molecules to penetrate the blood–brain barrier (BBB) without disrupting brain lipid levels. PCs target protein misfolding caused by mutations; this medication was first evaluated in Fabry disease, in which the use of α-galactosidase A inhibitors rescued the enzyme activity. PCs have also been evaluated in Gaucher disease, Pompe disease, gangliosidosis (GM1 and GM2), and MPS type II, IIIC, IVA, and IVB. Research continues to discover new PCs meeting specific criteria, including small size, cell permeability, and minimal side effects; many can cross the BBB safely. HSCT involves giving healthy hematopoietic stem cells to patients from various sources like bone marrow, peripheral blood, or umbilical cord blood, and relies on the fact that some lysosomal enzymes can be released into the blood stream and taken up by other cells through specific receptors. HSCT is proposed for several LSDs because it can provide a lifelong source of healthy cells expressing normal enzyme levels. These cells, found in circulating white blood cells and tissue-residing macrophages, can improve or stabilize clinical symptoms, extending life expectancy. In some cases, like MPS I, HSCT is the preferred treatment option and may be more effective if preceded by ERT administration. The effectiveness of GT and gene replacement methods for LSDs has been extensively demonstrated in animal models. The advent of genome editing tools like CRISPR/Cas9, and zinc finger nucleases (ZFN) allows for precise gene targeting and modification. Successful preclinical trials with AAV-mediated ZFN gene therapy have led to phase I/II clinical trials for MPS types I and II. While these trials have confirmed the safety of the technique, clinical observations underscore the need to enhance gene therapy strategies [169].

In many of the diseases involving the major intracellular pathways such as PI3K/AKT/mTOR, generically called mTORopathies, many therapies are underway [102]. Rapamycin and its analogues (rapalogues), oral or topical, have shown significant clinical efficacy in PROs or complex vascular anomalies, although to date the adverse side effects, the lack of specificity, and the incomplete suppression of mTOR targets may hinder their clinical use [102,168]. There are ongoing clinical trials targeting intracellular pathways like the PI3K/AKT/mTOR pathway, including sirolimus, miransertib (MK-7075) and alpelisib (BYL719) [168].

Autoimmune and, to some extent, autoinflammatory diseases are being treated, at least in some of their manifestations, with immunosuppressive therapies. In interferonopathies, the anti-IFN therapies may potentially be useful, although many questions have yet to be answered regarding the central role of IFN signaling in these diseases, where non-IFN-mediated pathways may be activated as well [170].

There are ongoing research efforts to develop novel therapies for these conditions. The variable responses to treatment make obvious the need for personalized therapeutic approaches in these rare diseases.

## 4. Conclusions

From the clinicians’ point of view, recognizing a disease or a syndrome may translate into better therapy. Early recognition and timely intervention are paramount to improve outcomes for affected individuals. Understanding the etiology of malformations may aid in the search for modifiable causes of abnormalities [1]. An improving knowledge of the cellular and molecular mechanisms bridging skeletal and neural development and inflammation in the developmental disorders will hopefully lead to designing new therapies. Increased awareness, timely recognition, and research could hopefully help the affected children and their families. There are ongoing research efforts aimed at understanding the cellular and molecular mechanisms underlying developmental disorders. Continuous research involving patients and families is very helpful in designing therapies and in improving outcomes. Development of clinical registries such as the Spanish Overgrowth Clinical Registry (SOGRI) may help conduct valuable data collections for advancing research [70]. Describing associated features and their possibly common mechanisms could also open the way for research in complex conditions [23].

## Figures and Tables

**Table 1:** Upper extremity changes in the context of macrocephaly.

Upper Extremity Changes	Syndromes	Head Enlargement Type	References
Polydactyly	VACTERL-H syndrome	MC	[28]
	Apert syndrome	MC	[29]
	Short-rib thoracic dysplasia 8	MC	[30]
	Bardet–Biedl syndrome	MC	[30]
	Gorlin syndrome	MC, HC, FB	[31,32,33,34,35]
	GCPS	MC	[36]
	Acro-callosal syndrome	MC	[37]
	Joubert syndrome 2	MC, FB	[38]
Oligodactyly	VACTERL-H syndrome	MC	[28]
Digit malposition	BRMUTD (First finger insertion)	MC, HC	[39]
Finger tapering	Turnpenny—Fry syndrome	FB	[40]
	Sifrim–Hitz–Weiss syndrome	MC	[41]
	Carey–Fineman–Ziter syndrome	MC	[42]
Camptodactyly	Osteopathia striata with cranial sclerosis	MC	[43]
		MC	[19]
	Sotos syndrome	MC	[19,44]
	Weaver syndrome	MC	[45,46]
	Cohen–Gibson syndrome	MC	[47]
	Rahman syndrome	MC	[48]
	Alcuraya–Kucinskas syndrome	MC	[38]
	Joubert syndrome 2	MC, FB	[49,50,51,52]
	Trichohepatoenteric syndrome	FB	[49,50,51,52]
Clinodactyly	Keipert syndrome	MC	[53]
	Osteopathia striata with cranial sclerosis	MC	[43]
	Peroxisome biogenesis disorder (Zellweger syndrome)	MC	[54,55]
	DDVIBA	MC	[56]
	Muenke craniosynostosis syndrome	MC	[57,58]
	Silver–Russel syndrome	MC	[59]
	SOFT syndrome	MC	[60]
	Larsen-like syndrome	MC	[61]
	CPRF	MC, FB	[48]
	Alcuraya–Kucinskas syndrome	MC	[62]
	Desmosterolosis	MC, FB	[42]
Brachydactyly	Congenital CMV infection	MC	[63,64]
	Robinow syndrome	MC	[65,66]
	Beta thalassemia	MC	[67,68]
	Pycnodysostosis	MC	[69]
	Pfeiffer syndrome	MC	[23]
	Gorlin syndrome	MC, FB	[31,32,33,34,35]
	Keipert syndrome	MC	[53]
	Simpson–Golabi–Behmel	MC	[70,71]
	Muenke craniosynostosis syndrome	MC	[57]
	SOFT syndrome	MC	[59]
	Temtamy syndrome	MC, FB	[72]
	Retinitis pigmentosa with or without skeletal anomalies	MC, FB	[42]
Syndactyly	VACTERL-H syndrome	MC	[28]
	Pfeiffer syndrome	MC	[23]
	Apert syndrome	MC, ME	[29]
	Gorlin syndrome	MC	[31,32,33,34,35]
	Simpson–Golabi–Behmel (second to third fingers)	MC	[73]
	GCPS (cutaneous syndactyly)	MC	[36]
Arachnodactyly	MDFPMR	MC, FB	[74,75,76]
	EDHHACC	MC, FB	[77]
Divergent fingers	Achondroplasia (trident hand)	MC	[3]
	CLOVES syndrome (wide spacing between digits)	MC, ME	[78,79]
Digital webbing	Pfeiffer syndrome	MC	[23]
	Apert syndrome	MC, ME	[29]
Polyphalangy	VACTERL-H syndrome (triphalangeal thumb)	HC	[28]
	Osteopathia striata with cranial sclerosis	MC	[43]
Broad terminal phalanges (spatulate fingers)	Keipert syndrome	MC	[53]
	Osteopathia striata with cranial sclerosis	MC	[43]
	Costello syndrome	MC	[80]
Phalangeal hypoplasia	Robinow syndrome	MC	[65,66]
	Joubert syndrome	MC	[30]
	Gorlin syndrome (First finger)	MC, HC, FB	[31,32,33,34,35]
	Smith–Kingsmore syndrome	MC, FB	[81]
	Simpson–Golabi–Behmel syndrome	MC	[73]
	Adams–Oliver syndrome	MC, HC	[82]
	Retinitis pigmentosa with or without skeletal anomalies	MC, FB	[42]
Phalangeal form changes	Mucopolysaccharidoses are bullet-shaped (proximal pointing)	MC, HC	[83,84,85,86]
	Muenke craniosynostosis syndrome (thimble-like middle phalanges)		
		MC	[57]
Brachymetacarpia (short metacarpal or metatarsal)	Noonan syndrome 2 (fifth finger)	MC	[87,88]
	Gorlin syndrome (fourth finger)	MC, HC, FB	[31,32,33,34,35]
	Pelger–Huet anomaly	MC	[89,90]
**Bone changes**			
Radial hypo/aplasia	VACTERL-H syndrome	HC	[28]
Radioulnar synostosis	VACTERL-H syndrome	HC	[28]
Ulnar deviation	Peroxisome biogenesis disorder (Zellweger syndrome)	MC	[54,55]
	Costello syndrome	MC	[80]
Bone sclerosis	Craniometaphyseal dysplasia AR	MC	[91]
Dysostosis multiplex	Mucopolysaccharidoses	MC, HC	[83,84,85,86]
Skeletal dysplasia	SPENCDI	MiC/MC/FB	[70,92,93,94,95]
	Singleton–Merten syndrome	MiC/MC/FB	
Enchondromas	SPENCDI	MiC/MC/FB	[92,93,94]
Acroosteolysis	Picnodysostosis	MC	[69]
	Cole–Carpenter syndrome	HC, FB	[96]
	Singleton–Merten syndrome	MiC/MC/FB	[95,97]
**Joint changes**			
Joint contractures	Mucopolysaccharidosis	MC, HC	[83,84,85,86]
	Gangliosidosis type I	MC	[8,98]
	Peroxisome biogenesis disorder (Zellweger syndrome)	MC	[54,55]
	Noonan syndrome type 2	MC	[87,88]
	L1 syndrome	HC	[99]
	Desmosterolosis	MC	[48]
	Carey–Fineman–Ziter	MC	[42]
	Singleton–Merten syndrome	MiC/MC, FB	[95,100,101]
Joint hypermobility	Osteogenesis imperfecta	MC	[96]
	Osteopathia striata with cranial sclerosis	MC, FB	[43]
	Pycnodysostosis	MC	[69]
	Pretzel syndrome	ME	[102]
	Cohen–Gibson syndrome	MC	[45,46]
	Tatton–Brown–Rahman syndrome	MC	[17]
	MDFPMR	MC, FB	[74,75,76]
Small joint arthritis	Cryopyrinopathies: CINCA/NOMID, Muckle–Wells etc.	FB, HC	[103]
	MVK deficiency	FB	[104]
	Beta-thalassemia	MC	[67,68]
Villonodular synovitis, multiple sites	Noonan syndrome	MC	[105,106]
Carpal tunnel syndrome	Mucopolysaccharidoses	MC, HC	[83,84,85,86]
**Nail changes**			
Nail dystrophy	Nail-patella syndrome	MC	[98]
	Robinow syndrome	MC	[65,66]
	Costello syndrome	MC	[80]
	Adams–Oliver syndrome	MC, HC	[82]
	SOFT syndrome	MC	[59]
	Primrose syndrome	MC	[61]
Single nail common to more digits	Apert syndrome	MC, FB, ME	[29]
**Skin changes**			
Thickened skin	Gangliosidosis type I	MC	[8,98]
Dry, hyperkeratotic skin	Cardiofaciocutaneous syndrome	MC	[107]
Wrinkled skin	Costello syndrome	MC	[80]
Pitted hands	Gorlin syndrome	MC, HC, FB	[31,32,33,34,35]
Deep palmar creases	Smith–Kingsmore syndrome	MC	[81]
Single palmar creases	Adams–Oliver 2 syndrome	MC	[108]
**Vascular, lymphatic, and other changes**			
Finger necrosis/amputation	Interferonopathies	MiC/MC, FB	[109,110,111,112]
Acrocyanosis	Congenital heart disease	MC	[113]
	Aicardi–Goutières	MC/MiC	[111,112]
Hand and feet edema	Mucopolysaccharidosis VI	MC	[83,84,85,86]
	Noonan syndrome	MC	[105,106]
Raynaud’s phenomenon	Neonatal SLE	MC	[114,115]
	Interferonopathies	MiC/MC	[109,110]
Soft tissue masses	PROS	MC, HC, FB	[31,32,33,34,35]

Legend: BRMUTD—brain malformation with or without urinary tract defects; CINCA/NOMID—Chronic infantile neurological, cutaneous, and articular syndrome/Neonatal-onset multisystem inflammatory disease; CMV—cytomegalovirus; CPRF—cleft palate, psychomotor retardation, and distinctive facial feature, DDVIBA—developmental delay with variable impairment and behavioral abnormalities; EDHHACC—Ectodermal dysplasia, hyperhidrotic with hypothyroidism and agenesis of the corpus callosus; FB—frontal bossing; GCPS—Greig cephalopolysyndactyly syndrome; HC—hydrocephalus; MC—macrocephaly; MDFPMR—macrocephaly, dysmorphic facies, and psychomotor retardation; MiC—microcephaly; MVK—mevalonate kinase deficiency; PROS—PI3KCA-associated overgrowth syndromes; SLE—Systemic lupus erythematosus; SOFT—short stature, onychodysplasia, facial dysmorphism and hypotrichosis; SPENCDI—spondyloenchondrodysplasia with immune features.

**Table 2:** Causes of macrocephaly with finger changes.

Disease	Inheritance	Causes/Mutations	Main Craniofacial and Hand/Finger Changes	References
**Acquired conditions**				
Rickets		Vitamin D deficiency	MC, FB, broad front, delayed closure of fontanelles, craniotabes, thickened wrists	[3]
Congenital infection		Cytomegalovirus infection	HC, cortical development abnormalities, brachydactyly	[63,64]
**Bone marrow disorders**				
Beta-thalassemia OMIM # 613985	AR (AD)	*HBB*	Prominent frontal and maxillary bones, sausage-like digits, small joints arthritis, fractures, signs of hypovitaminosis D	[67,68]
Congenital cyanotic heart diseases			MC with “hair-on-end” radiological skull changes, acrocyanosis	[113]
**Skeletal dysplasias**				
Achondroplasia OMIM # 100800	AD	*FGFR3*	MC, short fingers with divergent ring and middle fingers (trident hand)	[98]
Pycnodysostosis OMIM # 265800	AR	*CTSK*	MC, acro-osteolysis of the terminal phalanges, short fingers, joint hypermobility	[69]
Cole–Carpenter syndrome	AR	*P4HB*	HC, frontal bossing, craniosynostosis, ocular proptosis, frequent fractures, wide metacarpal and phalangeal epiphyses, cystic appearance, acro-osteolysis	[96]
VACTERL-H, VACTERL association, X-linked, with or without hydrocephalus; VACTERLX, OMIM # 314390	XLR	*SHH* signaling, *GLI3* *TBX-SALL4-SALL1-WNT* pathway *FGF8-FGF10* pathway	HC, radial hypo/aplasia, triphalangeal thumb, polydactyly, oligodactyly, syndactyly, radioulnar synostosis	[28,149,150]
Robinow syndrome OMIM # 268310	AR/AD	*ROR2*, *WNT5A*, *DVL1*, *DVL3*	MC, FB, limb shortening, brachydactyly, phalangeal and nail hypoplasia	[65,66]
Osteopathia striata with cranial sclerosis (OSCS) OMIM # 300373	XLD	*AMER1*	MC, FB, long, slender fingers, fifth finger clinodactyly, camptodactyly, finger contractures, duplicate phalanges, spatulate distal phalanges	[43]
Pfeiffer syndrome, OMIM # 101600	AD	*FGFR1* *FGFR2*	Cloverleaf skull, high forehead, syndactyly, brachydactyly, digital webbing	[23]
Apert syndrome OMIM # 101200	AD	*FGFR2*	ME, high, broad forehead, single nail common to digits 2 to 4, symmetric osseous and/or cutaneous syndactyly, polydactyly	[29]
Muenke craniosynostosis Syndrome (MNKES), OMIM # 602849	AD	*FGFR3*	MC, plagiocephaly, brachycephaly, premature suture closure, midface retrusion, hypertelorism, clinodactyly, brachydactyly, mild hand and feet anomalies	[23]
Craniometaphyseal dysplasia, autosomal recessive (CMDR) OMIM #218400	AR	*GJA1*	MC, coarse facial features, metacarpal and phalangeal sclerosis	[91]
Osteogenesis Imperfecta type X	AR	*SERPINH1*	MC, high forehead, triangular face, midface hypoplasia, hyperextensibility of the fingers	[151]
Keipert syndrome OMIM # 301026	XLR	*GPC4*	MC, hypertelorism, flat midface, prominent lips, brachydactyly, clinodactyly, broad terminal phalanges	[53]
Craniometaphyseal dysplasia, autosomal recessive (CMDR) OMIM #218400	AR	*GJA1*	MC, coarse facial features, metacarpal and phalangeal sclerosis	[91]
**Ciliopathies**				
Short-rib thoracic dysplasia 8 with or without polydactyly (SRTD8) OMIM # 615503	AR	*WDR60*	MC, polydactyly, skin changes	[30]
Bardet–Biedl syndrome OMIM # 617119	AR	*IFT74*	MC (or MiC), polydactyly	[30]
Joubert syndrome OMIM # 213300	AR	*INPP5E*	MC, prominent forehead, high rounded eyebrows, missing digital phalanges	[30]
**Inherited metabolic disorders**				
Mucopolysaccharidoses MPS I (Hurler syndrome) OMIM # 607014 MPS II (Hunter syndrome), OMIM # 309900 MPS VI (Maroteaux-Lamy), OMIM # 253200 MPS VII (Sly syndrome), OMIM # 253220	MPS I: AR, MPS II: X-linked, MPS VI: AR, MPS VII: AR	MPS I: *IDUA* MPS II: *IDS* MPS VI: *ARSB*, MPS VII: *GUSB*	MPS I: large head with bulging frontal bones (MC, HC), carpal tunnel syndrome with weakness in the hand and fingers, phalanges are bullet-shaped with proximal pointing of the second to fifth metacarpals. MPS II: MC, claw hands, stiffness, joint contractures, carpal tunnel syndrome, joint hypermobility, dysostosis multiplex. MPS VI: coarse dysmorphic features, HC, edema of the hands and feet, dysostosis multiplex. MPS VII (mild form): MC, mild craniofacial dysmorphism, dysostosis multiplex	[83,84,85,86]
Gangliosidosis type I OMIM # 230500	AR	*GLB1*	MC, claw hands, thickened subcutaneous tissues	[8,98]
Alpha-mannosidosis type I (Hurler-like disease) OMIM # 248500	AR	*MAN2B1*	HC, large head with prominent forehead, dysostosis multiplex	[152]
Peroxisome biogenesis disorder (cerebrohepatorenal/ Zellweger syndrome) OMIM # 214100	AR	*PEX1*, *PEX3*, *PEX6*, *PEX16*, *PEX2*, *PEX12*, *PEX14*,	MC (or MiC), dysmorphic features (large anterior fontanel, prominent high forehead), finger flexion; long fingers, deviated to the ulnar side, the thumbs were not held in apposition	[54,55]
**RASopathies**				
Costello syndrome OMIM # 218040	AD	*HRAS*	Coarse facies, wrinkled skin, splayed spatulate fingers, abnormal nails, ulnar deviation	[80]
Cardio-facio-cutaneous syndrome OMIM # 115150	AD	*BRAF*, *MAP2K1*, *MAP2K2*, *KRAS*	Joint contractures, dry, hyperkeratotic scaly skin, ulnar deviation, deep palmar creases	[107]
Noonan syndrome (NS) NS 1, OMIM # 163950 NS 3, OMIM # 609942 NS 4, OMIM # 610733 NS 5, OMIM # 611553 NS 6, OMIM # 613224	AD	*PTPN11*, *KRAS*, *SOS1*, *RAF1*, *NRAS*,	Broad forehead, dolichocephaly, polyarticular pigmented villonodular synovitis, peripheral lymphedema	[105,106]
Noonan syndrome (NS) 2 OMIM # 605275	AR	*LZTR1*	Broad forehead, fifth brachymetapody, arthrogryposis	[87,88]
**Overgrowth syndromes**				
Gorlin–Goltz syndrome/ Gorlin syndrome/ [Nevoid basal cell carcinoma syndrome (NBCCS)] OMIM # 109400	AD	*PTCH1*	MC/ relative MC, mild HC, FB, pitted hands, brachydactyly, short fourth metacarpal, polydactyly, 2–3 syndactyly, short thumb terminal phalanx	[31,32,33,34,35]
PTEN hamartoma tumor syndrome: Cowden’s syndrome, Bannayan–Riley–Ruvalcaba syndrome Lhermitte–Duclos syndrome OMIM # 158350	AD	*PTEN*	MC/ME, HC, asymmetric soft-tissue masses, increased fat deposition, enlarged vessels MC/ME, HC, cerebellar signs, papilledema	[133] [134]
Smith–Kingsmore syndrome (SKS), Minds syndrome OMIM # 616638	AD	*MTOR*	MC, FB, tall forehead, midface hypoplasia, short proximal and distal phalanges, deep palmar creases	[81]
CLOVES syndrome, OMIM # 612918 Proteus syndrome, OMIM # 176920 MCAP syndrome, OMIM # 602501 MPPH syndrome 1 OMIM # 603387	AD, somatic mutations, arise randomly in one cell during embryonic development	PI3K/AKT/mTOR pathway (*CCND2*, *PIK3R2*, *AKT3*, *PIK3CA*, *MCC*, *NSD1*)	MC, ME and capillary malformations, asymmetric overgrowth of the extremities, wide spacing between digits, lymphatic anomalies	[78,79]
Sotos syndrome OMIM # 117550	AD	*NSD1*	MC, high broad forehead, long face, prominent chin, advanced bone age, camptodactyly	[19]
Weaver syndrome OMIM # 277590	AD	*EZH2*, *NSD1*	MC, camptodactyly of the fingers and/or toes, hyperextensibility of the fingers, finger contractures, thin, deep-set nails, boutonniere deformity in adults	[19,44,153]
Malan syndrome OMIM # 277590	AD	*NFIX*	MC, long and narrow triangular face, prognathia, aortopathy, advanced bone age, scoliosis, long hands, ID	[20]
* Polyhydramnios, megalencephaly, and symptomatic epilepsy syndrome, Pretzel syndrome (PMSE) OMIM # 611087	AR	*STRAD-alpha* (*LYK5*)	ME, cognitive delay, hyperextensible fingers	[102]
Simpson–Golabi–Behmel syndrome (SGBS1) OMIM # 312870	X-linked	*GPC3*, *GPC4*	MC, pre- and postnatal overgrowth, coarse facies, index finger hypoplasia, syndactyly second to third fingers, brachydactyly, broad hands, polydactyly	[70,71,73]
Cohen–Gibson syndrome (COGIS) OMIM # 617561	AD	*EED*	MC, broad forehead, long fingers, broad thumbs, camptodactyly, joint laxity of the small joints of the hand	[45,46]
Rahman syndrome (RMNS) OMIM # 617537	AD	*HIST1H1E* (*H1-4*)	Increased height and/or head circumference early in life, camptodactyly	[47]
Tatton–Brown–Rahman syndrome (TBRS) OMIM #615879	AD	*DNMT3A*	MC, round facies, bushy eyebrows, prominent maxillary incisors, joint hyperlaxity	[17]
Intellectual developmental disorder with hypertelorism and distinctive facies OMIM # 618147	AD	*CCNK*	MC, high anterior hairline, tapered fingers	[154]
Brain malformations with or without urinary tract defects (BRMUTD) OMIM # 613735	AD	*NFIA*	MC, ventriculomegaly, overgrowth, bilateral proximally placed first fingers	[39]
Developmental Delay with Variable Intellectual Impairment and Behavioral Abnormalities (DDVIBA) OMIM # 618430	AD	*TCF20*	MC, brachycephaly, FB, Tapering fingers, fifth finger clinodactyly	[56]
**Other inherited causes**				
L1 syndrome OMIM # 307000	XLR	*L1CAM*	HC, arthrogryposis, adducted thumbs, developmental delay	[99]
Adams–Oliver syndrome (AOS1) OMIM # 100300	AD/ AR	*NOTCH1*, *ARHGAP31*, *DOCK6*, *EOGT*, *DLL4*, or *RBPJ*	Encephalocele, ventriculomegaly, slight ventricular dilation, periventricular leukomalacia, short or missing phalanges, dysplastic or absent nails	[82]
Adams–Oliver syndrome-2 (AOS2) OMIM # 614219	AR	*DOCK6*	MC (or MiC), mild facial dysmorphism, low hair line, shortened digits, single palmar creases	[108]
Macrocephaly, dysmorphic facies, and psychomotor retardation (MDFPMR) OMIM # 617011	AR	*HERC1*	MC, FB, somatic overgrowth apparent at birth, seizures, joint laxity, and long fingers; large hands with arachnodactyly	[74,75,76]
Silver–Russell Syndrome OMIM # 180860	*hypomethylation on ch. 11p15.5 or maternal UPD* *for ch. 7*	*IGF2*, *CDKN1C*, *PLAG1*, *HMGA2*, *H19*	FB or prominent forehead, fifth finger clinodactyly	[58]
Turnpenny–Fry syndrome OMIM # 618371	AD	*PCGF2*	FB, short tapering fingers	[40]
Greig cephalopolysyndactyly syndrome (GCPS)OMIM # 175700	AD	*GLI3*	MC, high, prominent forehead, preaxial polydactyly, abnormally wide thumb or big toe, cutaneous syndactyly	[36]
Short stature, onychodysplasia, facial dysmorphism, and hypotrichosis (SOFT syndrome)OMIM # 614813	AR	*POC1A*	MC (present during early childhood), protruding forehead, short rectangular fingers and hypoplastic fingernails, clinodactyly, brachydactyly	[59]
Acro-callosal Syndrome (ACLS), Joubert syndrome 12, OMIM # 200990	AR	*KIF7*	MC, prominent forehead, postaxial polydactyly of the hands, and preaxial polydactyly of the feet	[37]
Intellectual developmental disorder, X-linked syndromic, Cabezas type OMIM # 300354	XLR	*CUL4B*	MC/relative MC, short thumbs, and little fingers with adduction, brachydactyly	[155]
Ectodermal Dysplasia, Hypohidrotic, with Hypothyroidism and Agenesis of The Corpus Callosum OMIM # 225040	may be XL	may represent a contiguous gene syndrome	MC, FB, long slender fingers	[77]
Primrose syndrome (PRIMS) OMIM # 259050	AD	*ZBTB20*	MC, dystrophic/abnormal fingernails, and toenails	[61]
Larsen-like syndrome OMIM # 608545	Isolated cases	location: 6p25	MC, brachycephaly, prominent forehead, cylindrical fingers, clinodactyly (fourth and fifth fingers)	[60]
Cleft palate, psychomotor retardation, and distinctive facial feature (CPRF) OMIM # 616728	AD	*KDM1A*	MC (in some patients), brachycephaly, FB, tapered fingers, fifth finger clinodactyly, short thumbs	[156]
Chromosome 5p13 duplication syndrome OMIM # 613174	Isolated cases	microduplications 5p13	MC, turricephaly, FB, broad forehead, large hands, long fingers	[157]
Phelan–McDermid syndrome (PHMDS) OMIM # 606232	AD	*SHANK3*	MC, dolichocephaly, asymmetric face, dysplastic toenails	[158]
Sifrim–Hitz–Weiss syndrome (SIHIWES) OMIM # 617159	AD	*CHD4*	MC, trigonocephaly, coarse facies, tapered fingers, fusion of the wrist bones	[41]
Alkuraya–Kucinskas syndrome (ALKKUCS) OMIM # 617822	AR	*KIAA1109*	MC, plagiocephaly, overlapping fingers, camptodactyly, clenched hands adducted thumbs, clinodactyly	[48]
Desmosterolosis OMIM # 602398	AR	*DHCR24*	MC relative (sometimes microcephaly), FB, arthrogryposis, fifth finger clinodactyly,	[62]
Wiedemann–Rautenstrauch syndrome (WDRTS) OMIM #264090	AR	*POLR3A*	MC/relative MC, FB, triangular face, long fingers, large hands	[159]
Joubert syndrome 2 (JBTS2) OMIM # 608091	AR	*TMEM216*	MC, dolichocephaly, FB, postaxial polydactyly, camptodactyly	[38]
Temtamy syndrome (TEMTYS) OMIM #218340	AR	*C12ORF57*	MC, FB, long face, brachydactyly (second to fifth fingers), bulbous thumbs	[72]
Carey–Fineman–Ziter syndrome (CFZS1) OMIM # 254940	AR	*MYMK*	MC, (sometimes microcephaly), plagiocephaly, tapering fingers, distal contractures	[42]
Retinitis pigmentosa with or without skeletal anomalies OMIM #250410	AR	*CWC27*	MC (in some patients), FB, brachydactyly, shortening of distal phalanges	[160]
Pelger–Huet anomaly OMIM #169400	AD	*LBR*	MC with prominent forehead, short metacarpals in several fingers	[89,90]
Fragile X syndrome, OMIM # 300624	XLD	*FMR1*	MC, coarse facies, large forehead, long face prominent jaw, hyperextensibility finger joints, dermatoglyphic findings, double-jointed thumbs	[161,162,163,164]
Spinocerebellar ataxia, autosomal recessive 20, OMIM # 616354	AR	*SNX14*	Relative MC, clinodactyly, camptodactyly, brachydactyly	[165]
**Autoimmune**				
Systemic lupus erythematosus			MC in 8% neonatal SLE, HC; Raynaud’s phenomenon, vasculitis, rashes	[114,115]
Juvenile idiopathic arthritis			HC (rarely); small joints arthritis	[140]
Systemic sclerosis			HC (rarely), Raynaud’s phenomenon, sclerodactyly	[140].
**Autoinflammatory**				
Cryopyrinopathies [CINCA/NOMID (OMIM # 607115), Muckle-Wells (OMIM # 191900), FCAS (OMIM # 120100)]	AD	*NLRP3*	Urticarial-like rashes, aseptic meningitis, FB, MC, oligoarthritis	[103]
* Mevalonate kinase deficiency (MVK, mevalonic aciduria, hyper IgD syndrome) OMIM # 610377	AR	*MVK*	FB, dolichocephaly, triangular facies; rash, edema and arthralgia may occur during febrile crisis	[104]
Spondyloenchondrodysplasia with immune features OMIM # 607944	AR	*ACP5*	Cranio-facial deformities, hand anomalies, enchondromas	[92,93,94]
Aicardi–Goutières syndrome (AGS) AGS1: OMIM # 225750 AGS2: OMIM # 610181 AGS3: OMIM # 610329 AGS4: OMIM # 610333 AGS5: OMIM # 612952 AGS6: OMIM # 615010 AGS7: OMIM # 615846 AGS8: OMIM # 619486 AGS9: OMIM # 619487	AD (for several cases with *TREX1gene mutation*), AR	*TREX1*, *RNASEH2B*, *RNASEH2C*, *RNASEH2A*, *SAMHD1*, *ADAR*, *IFIH-1*, *LSM11*, *RNU7-1*	MiC/MC, pseudo-TORCH syndrome, dysmorphic features; acrocyanosis, autoamputation of the fingers, chilblain-like lesions	[109,110,111,112]
Singleton–Merten syndrome (SGMRT) OMIM # 182250	AD	*IFIH1*	Broad forehead, high hairline, acro-osteolysis, skeletal dysplasia, aortic calcifications, psoriasis, glaucoma	[95,97]
* Atypical Singleton–Merten syndrome SGMRT2, OMIM # 616298	AD	*DDX58*	Similar to SGMRT1, arthritis of the hands, metacarpophalangeal contractures; possibly calcified ligaments of the interphalangeal and metacarpophalangeal joints, mild distal erosions	[95,100,101]
* Tenorio syndrome OMIM # 616260	AD	*RNF125*	MC, overgrowth, large forehead, mild HC, Sjogren’s syndrome features	[70]
USP18 deficiency, OMIM # 617397	AR	*USP18*	Pseudo-TORCH syndrome 2; HC, brain malformation, metaphyseal X-ray changes resembling intrauterine infections	[142,144,166,167]
* Trichohepatoenteric syndrome (THES) THES1: OMIM # 222470 THES2: OMIM # 614602	AR	*TTC37 (SKIC3)* *SKIV2L (SKIC2)*	Prominent forehead and cheeks, broad nasal root, trichorrhexis nodosa, skin changes, diarrhea; café-au-lait spots on the lower limbs, camptodactyly	[49,50,51,52,142]

Legend: * conditions that may exhibit characteristics or features overlapping with multiple categories, due to shared characteristics or complexities in classification. AD—autosomal dominant; AGS—Aicardi–Goutières syndrome, AR—autosomal recessive, ch—chromosome, CINCA/NOMID—chronic infantile neurologic cutaneous articular syndrome/neonatal-onset multisystem inflammatory disease, CLOVES syndrome—Congenital Lipomatous Overgrowth, Vascular Malformations, Epidermal Nevis, Spinal/Skeletal Anomalies/Scoliosis, DADA2—adenosine deaminase-2 deficiency, FB—frontal bossing, FCAS—familial cold-induced autoinflammatory syndrome, HC—hydrocephalus, GCPS—Greig cephalopolysyndactyly syndrome, ID—intellectual disability, MC—macrocephaly, MiC—microcephaly, MCAP—megalencephaly-capillary malformation syndrome, ME—megalencephaly, MPPH—megalencephaly–polymicrogyria–polydactyly–hydrocephalus syndrome, MPPM—megalencephaly–polymicrogyria–pigmentary mosaicism, MPS—Mucopolysaccharidosis, mTOR—mammalian target of rapamycin, OMIM—Online Mendelian Inheritance in Man, PTEN—phosphatase and tensin homologue deleted on chromosome 10, SGMRT—Singleton–Merten syndrome, UPD—uniparental disomy, XL—X-linked disorder, XLD—X-linked dominant disorder, XLR—X-linked recessive disorder.; VACTERL-H: vertebral, anal, cardiac, trachea–esophageal fistula, esophageal/duodenal atresia, renal, limb, hydrocephalus; PHENOS–skin pigmentation, small head, small eyes, nervous system, otology, short stature.

## Supplementary Materials

The following supporting information can be downloaded at: https://www.mdpi.com/article/10.3390/ijms25105567/s1.

## Abbreviations

ACP5: Encoding the tartrate-resistant acid phosphatase 5
AD: Autosomal dominant
AOS: Adams–Oliver syndrome
AR: Autosomal recessive
BMP: Bone morphogenetic protein
BRMUTD: Brain malformations with or without urinary tract defects (BRMUTD)
CFZS1: Carey–Fineman–Ziter syndrome
CFC: Cardiofaciocutaneous syndrome
CINCA/NOMID: Chronic infantile neurologic cutaneous articular syndrome/neonatal-onset multisystem inflammatory disease
GCPS: Greig cephalopolysyndactyly syndrome
CPRF: Cleft palate, psychomotor retardation, and distinctive facial feature
CLAPO syndrome: Capillary malformation of the lower lip, lymphatic malformation of the face and neck, asymmetry, and partial/generalized overgrowth
CLOVES syndrome: Congenital Lipomatous Overgrowth, Vascular Malformations, Epidermal Nevis, Spinal/Skeletal Anomalies/Scoliosis
CMDR: Craniometaphyseal dysplasia, autosomal recessive
CMV: Cytomegalovirus
CNS: Central nervous system
CNVs: Copy number variations
COGIS: Cohen–Gibson syndrome
DADA2: Adenosine deaminase-2 deficiency
DDVIBA: Developmental delay with variable impairment and behavioral abnormalities
EDHHACC: Ectodermal dysplasia, hyperhidrotic with hypothyroidism and agenesis of the corpus callosus
EGF: Epidermal growth factor
ERT: Enzyme replacement therapy
FB: Frontal bossing
FCAS: Familial cold-induced autoinflammatory syndrome
FGFR: Fibroblast growth factor receptors
GAGs: Glycosaminoglycans
GT: Gene therapy
HC: Hydrocephalus
HMEG: Hemimegaloencephaly
HSCT: Hematopoietic stem cell transplantation
ID: Intellectual disability
IGF-I: Insulin-like growth factor I
IHH-PTHrP: IHH (Indian Hedgehog), which stimulates PTH-related protein pathway
JIA: Juvenile idiopathic arthritis
LSDs: Lysosomal storage disorders
MC: Macrocephaly
MCAP: Macrocephaly-capillary malformation
ME: Megalencephaly
MDFPMR: Macrocephaly, dysmorphic facies, and psychomotor retardation
MiC: Microcephaly
MNKES: Muenke craniosynostosis syndrome
MPPH: Megalencephaly-polymicrogyria-polydactyly-hydrocephalus syndrome
MPPM: Megalencephaly-polymicrogyria-pigmentary mosaicism
MPS: Mucopolysaccharidoses
mTOR: Mammalian target of rapamycinNGS: next-generation sequencing
NS: Noonan syndrome
OMIM: Online Mendelian Inheritance in Man
PBD: Peroxisome Biogenesis Disorders
PCs: Pharmacological chaperones
PHTS: PTEN Hamartoma-tumor syndrome
PRAAS: Proteasome-associated autoinflammatory syndromes
PROS: PIK3CA-related overgrowth spectrum
PTEN: Phosphatase and tensin homologue deleted on chromosome 10
SAVI: STING associated vasculopathy with onset in infancy
SGMRT: Singleton-Merten syndrome
SHH: Sonic hedgehog (pathway)
SIHIWES: Sifrim–Hitz–Weiss syndrome
SOGRI: Spanish Overgrowth Clinical Registry
SPENCD: Spondyloenchondrodysplasia
SRT: Substrate reduction therapy
SRTD8: Short-rib thoracic dysplasia 8 with or without polydactyly
STING: Stimulator of Interferon Genes
TBRS: Tatton–Brown–Rahman syndrome
THES: Trichohepatoenteric syndrome
TSC1, TSC2: Tuberous sclerosis complex 1, Tuberous sclerosis complex 2
TSH: thyroid-stimulating hormone
UPD: Uniparental disomy
VEGF: Vascular endothelial growth factor
XL: X-linked disorder
XLD: X-linked dominant disorder
