# Supplementary material for: Macrocephaly and Finger Changes: A Narrative Review
Source: Int J Mol Sci. 2024 May 20;25(10):5567. doi: 10.3390/ijms25105567 (PMC11122644; doi:10.3390/ijms25105567)
Supplement: Supplementary file 1 [file ijms-25-05567-s001.zip › ijms-2939762-supplementary.pdf]

Supplementary Table S1. Mean normal head circumference by gestational age [2].

Gestational age (weeks)	Head circumference in cm (boys/girls)
24	22.7/22.1
26	24.6/23.8
28	26.3/25.7
30	28.3/27.7
32	30.1/29.6
34	31.9/31.5
36	33.5/33.1
38	34.6/34.0
40	35.2/34.6

Supplementary Table S2. Causes of macrocephaly [3,5,11,14–16].

<table>
<tr><th>Affected system (hypertrophic or hyperplastic structure)</th><th>Type of insult</th><th>Categories of diseases</th><th colspan="2">Examples</th></tr>
<tr><td rowspan="12">Increased brain size (megalencephaly)</td><td rowspan="12">Anatomic</td><td rowspan="9">Neurocutaneous diseases</td><td colspan="2">Neurofibromatosis type I, OMIM # 162200</td></tr>
<tr><td colspan="2">Legius syndrome, OMIM # 611431</td></tr>
<tr><td colspan="2">Cardiofaciocutaneous syndrome 1, OMIM # 115150</td></tr>
<tr><td colspan="2">Costello syndrome, OMIM # 218040</td></tr>
<tr><td colspan="2">LEOPARD syndrome 2, OMIM # 611554</td></tr>
<tr><td colspan="2">Noonan syndrome 2, OMIM # 605275< Noonan syndrome 5, OMIM # 611553</td></tr>
<tr><td colspan="2">Nevoid Basal Cell Carcinoma Syndrome 1 (Gorlin-Goltz syndrome), OMIM # 109400</td></tr>
<tr><td colspan="2">Sturge-Weber syndrome, OMIM # 185300</td></tr>
<tr><td colspan="2">Tuberous sclerosis, OMIM # 191100</td></tr>
<tr><td rowspan="3">Overgrowth syndromes</td><td rowspan="3">Syndromes with skin or vascular features</td><td>CLOVES syndrome, OMIM # 612918</td></tr>
<tr><td>Klippel-Trenaunay syndrome, OMIM 149000</td></tr>
<tr><td>Proteus syndrome</td></tr>
</table>

				Megalencephaly-Polymicrogyria-Pigmentary Mosaicism syndrome
			Syndromes without skin or vascular features	Beckwith-Wiedemann syndrome
				Cowden syndrome 1, OMIM # 158350
				Pretzel syndrome, OMIM # 611087
				Simpson-Golabi-Behmel syndrome, OMIM # 312870
				Sotos syndrome, OMIM # 117550
				Weaver syndrome, OMIM # 277590
				Macrocephaly, dysmorphic facies, and psychomotor retardation (MDFPMR), OMIM # 617011
		Hamartoma Tumor Syndrome, OMIM # 158350		
		Hemimegalencephaly (HMEG),		
		Megalencephaly-Capillary Malformation-Polymicrogyria syndrome, OMIM # 602501		
		Smith-Kingsmore Syndrome, OMIM # 616638		
		Fragile X syndrome, OMIM # 300624		
		Lujan-Fryns syndrome, OMIM # 309520		
		Opitz–Kaveggia syndrome, OMIM # 305450		
	**Metabolic**	Organic acid disorders	Glutaric acidemia type I (OMIM # 231670), type II (OMIM # 231680)	
			D2-hydroxyglutaric aciduria, OMIM # 600721	
		Lysosomal storage diseases	Mucopolysaccharidosis	
			Gangliosidosis	
			Krabbe disease, OMIM # 245200	
		Peroxisome biogenesis disorder	Zellweger/ cerebrohepatorenal Syndrome, OMIM # 614859	
		Leukoencephalopathies	Alexander disease, OMIM # 203450	
			Canavan disease, OMIM # 271900	
			Megalencephalic Leukoencephalopathy with Subcortical Cysts, OMIM # 604004	
			Vanishing White Matter Disease 5, OMIM # 620315	

**CSF**	Hydrocephalus	Obstructive	Brain tumours
			Chiari malformation, OMIM # 118420
			Dandy-Walker syndrome malformation, # 220200
			Aqueductal stenosis
		Communicating	Deficient resorption of CSF
			Altered blood circulation within the brain
	Benign enlargement of the subarachnoid space		
**Blood**	Haemorrhage		
**Bone**	Bone marrow	Thalassemia	
	Bone disorders	Rickets	
		Skeletal dysplasia	Achondroplasia, OMIM # 100800
			Cleidocranial dysostosis, OMIM # 119600
			Cole-Carpenter syndrome 1, OMIM # 112240 and Cole-Carpenter syndrome 2, OMIM # 616294
			Craniometaphyseal dysplasia, OMIM # 123000
			Hyperphosphatasia, OMIM # 616809
			Metaphyseal dysplasia, OMIM # 250400
			Osteopetrosis AR 1, OMIM # 259700
			Osteopathia striata with cranial sclerosis, OMIM # 300373
			Osteogenesis imperfecta type III, # 259420
			Robinow syndrome 1, OMIM # 268310
